# Gender representation among speakers at the Japanese Society of Anesthesiologists meetings: A retrospective analysis

**DOI:** 10.1371/journal.pone.0320398

**Published:** 2025-03-28

**Authors:** Michiko Kinoshita, Yoko Sakai, Katsuya Tanaka

**Affiliations:** 1 Department of Anesthesiology, Tokushima University Hospital, Tokushima-shi, Tokushima, Japan; 2 Division of Anesthesiology, Tokushima University Hospital, Tokushima-shi, Tokushima, Japan; Emory University, UNITED STATES OF AMERICA

## Abstract

**Purpose:**

This study investigates the gender distribution of speakers at the Japanese Society of Anesthesiologists (JSA) annual and branch meetings of the Japanese Society of Anesthesiologists.

**Methods:**

We examined the gender of speakers in sessions at both JSA annual and branch meetings. We also verified the speakers’ Japanese medical licensure status and years of qualification.

**Results:**

We analyzed 383 sessions from JSA annual meetings between 2019 and 2024, which included 827 speaker slots. Of them, 679 (82.1%) were men and 148 (17.9%) were women. Women were significantly underrepresented in sessions with fewer speaker slots (chi-square test, p =  0.006; trend test, p <  0.001). Furthermore, sessions were frequently composed entirely of men: 73.1% of all sessions and 44.3% of panel presentations were solely male participants. Among the subspecialties, female representation was high in obstetric anesthesia (36.8%) and pediatric anesthesia (31.8%) but low in cardiovascular anesthesia (6.3%). Among 508 speakers with confirmed Japanese medical licenses, 425 (83.7%) were men, and 83 (16.3%) were women, with no significant differences in gender distribution based on the year of licensure (Fisher’s exact test, p =  0.968; trend test, p =  0.463). Additionally, we examined 104 sessions from JSA branch meetings between 2019 and 2023, comprising 176 speaker slots. Of them, 147 (83.5%) were men and 29 (16.5%) were women. There was no significant difference in gender distribution among branch meetings across different regions (p =  0.984).

**Conclusion:**

These findings underscore the need for proactive measures to promote gender diversity in Japan’s anesthesiology field.

## Introduction

The proportion of women in the Japanese Society of Anesthesiologists (JSA) has been steadily increasing, rising from 25.6% in 2001 to over 30% in 2007 and surpassing 40% by 2021. Previous research has shown an increase in women being the first authors of peer-reviewed anesthesiology articles [1]. Since 2010, women have contributed to approximately 25% of such publications from Japan [1]. However, women remain underrepresented in anesthesiology leadership roles in Japan, highlighting the need for strategies that promote gender diversity [1].

Academic conference presentations are crucial for career advancement, showcasing achievements, and enhancing reputations [2]. Women speakers at these conferences act as role models for trainees and colleagues, inspiring greater participation by younger women in the field [3,4]. Previous studies have examined the gender of speakers at the annual meetings of the American Society of Anesthesiologists (ASA) [5], Canadian Anesthesiologists’ Society (CAS) [6], and Society of Cardiovascular Anesthesiologists (SCA) [7], indicating the need to support women’s representation. While conducting gender disparity surveys is crucial to achieving speaker balance at conferences [8], no studies have examined this issue at JSA meetings.

Thus, this study aimed to investigate the gender distribution of speakers at JSA annual and branch meetings. Given the JSA’s advocacy for advancing women in academia [9], this study’s findings can provide valuable insights to support their efforts to improve gender diversity in Japanese anesthesiology.

## Materials and methods

This retrospective cross-sectional study did not require approval from an Institutional Ethics Board as it involved a bibliometric analysis using publicly available data.

Detailed information on the JSA annual and branch meetings was obtained from the JSA members’ website on April 15, 2024, with data publicly accessible through each meeting’s official website. The study period covered the JSA annual meetings from 2019 to 2024 and branch meetings up to 2023, depending on data availability. Only solicited sessions were included in the analysis, whereas poster presentations, problem-based learning discussions, lectures for JSA-certified anesthesiologists, academic award commemorative presentations, and sponsored lectures were excluded. For annual meetings, we also examined the composition of program committee members.

The gender of the speakers was determined based on their names, following methodologies used in previous studies [1,10,11]. In cases where name-based identification was challenging, gender was confirmed through publicly available photos on the Internet. Additionally, we examined whether each speaker held a Japanese medical license and identified their year of licensure. This information was obtained from the medical doctor qualification verification search website operated by the Ministry of Health, Labour, and Welfare [12]. This website also publicly discloses the gender information of licensed medical doctors. Further verification was conducted through additional Internet searches for speakers with identical or previous names.

Data are presented as numbers (percentages) or medians (interquartile ranges). Ratios of binary data were compared using the chi-square test or Fisher’s exact test when cell counts were five or fewer. Trends in binary data ratios were analyzed using the Cochran-Armitage trend test. The median values between the two groups were compared using the Mann–Whitney U test. Correlations between the two variables were assessed using Spearman’s rank correlation coefficients. All p-values were two-sided, and values less than 0.05 were considered statistically significant. Statistical analyses were conducted using EZR (Saitama Medical Center, Jichi Medical University, Saitama, Japan), a graphical user interface for R version 4.1.3 (The R Foundation for Statistical Computing, Vienna, Austria) that provides statistical functions commonly used in biostatistics [13].

## Results

We analyzed 383 eligible sessions from the JSA annual meetings between 2019 and 2024. In total, 827 speaker slots were identified for individual presentations and panels, comprising 679 men (82.1%) and 148 women (17.9%). Gender distribution showed no significant differences across the years (chi-square test, p =  0.324; trend test, p =  0.787) or for speaker affiliations (p =  0.376). Analysis of gender distribution by session size revealed that women were significantly underrepresented in sessions with fewer speakers (chi-square test, p =  0.006; trend test, p <  0.001) (Table 1).

**Table 1:** Gender distribution of speaker slots at the JSA annual meetings, 2019–2024.

	Men no. (%)	Women no. (%)	p-value	
			Chi-square test	Trend test
**Overall**	679 (82.1)	148 (17.9)		
**Year**			0.324	0.787
2019	137 (85.6)	23 (14.4)		
2020	35 (85.4)	6 (14.6)		
2021	115 (76.2)	36 (23.8)		
2022	113 (80.7)	27 (19.3)		
2023	129 (83.8)	25 (16.2)		
2024	150 (82.9)	31 (17.1)		
**Affiliation**			0.376	
Japan	603 (82.6)	127 (17.4)		
Overseas	76 (78.4)	21 (21.6)		
**Session size**			0.006	< 0.001
One speaker	222 (88.1)	30 (11.9)		
2–3 speakers panel	43 (87.8)	6 (12.2)		
4–5 speakers panel	341 (79.1)	90 (20.9)		
6–8 speakers panel	73 (76.8)	22 (23.2)		

Session size is based on the number of speaker slots.

**Fig 1:**
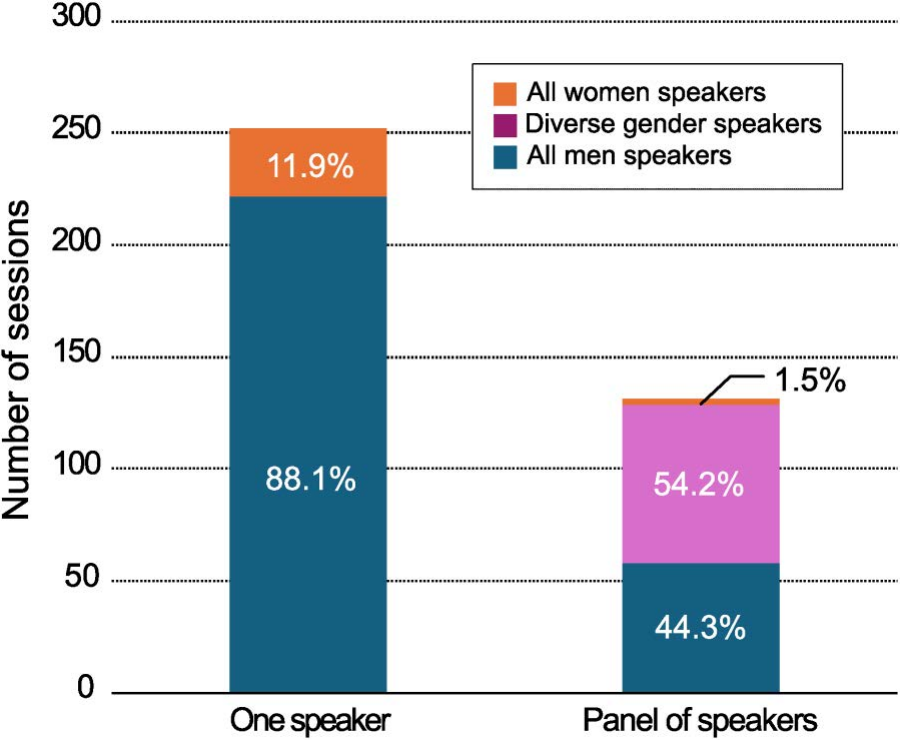
Gender composition of sessions at the JSA annual meetings.

Of the 383 sessions, 280 (73.1%) consisted entirely of male speakers. In the panel presentations, 58 of 131 (44.3%) participants were male (Fig 1).

**Fig 2:**
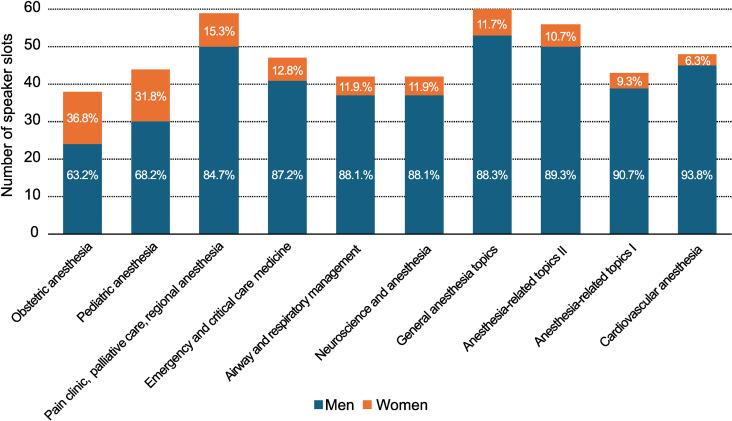
Gender representation across subspecialties at the JSA annual meetings.

Among the subspecialties, women’s representation showed notable variation: higher in obstetric (36.8%) and pediatric anesthesia (31.8%), but lower in cardiovascular anesthesia (6.3%) (Fig 2).

The following categories are classified by the JSA. General anesthesia topics cover broad issues such as manpower challenges, the management of operating rooms and medical facilities, risk management, medical ethics, safety protocols, education, and the history of anesthesiology. Whereas, anesthesia-related topics I focus on perioperative management, including pre- and postoperative evaluations, intraoperative complications, physiology, pharmacology, anesthesia-related equipment, fluid management, and statistical analyses. And, anesthesia-related topics II delve into the development of medical devices, monitoring techniques, anesthesia practices outside the operating room, day surgery, and subspecialty anesthesia, including ophthalmology, head and neck surgery, orthopedics, and anesthesia for elderly patients.

Over the six-year study period, we identified 655 unique speakers, comprising 537 (82.0%) men and 118 (18.0%) women. Although some individuals were presented multiple times, the gender distribution did not significantly differ based on presentation frequency (chi-square test, p =  0.403; trend test, p =  0.864) (Table 2).

**Table 2:** Gender distribution of individual speakers at the JSA annual meetings, 2019–2024.

	Men no. (%)	Women no. (%)	p-value	
			Chi-square/Fisher’s exact test	Trend test
**Overall**	537 (82.0)	118 (18.0)		
**Affiliation**			0.332	
Japan	467 (82.7)	98 (17.3)		
Overseas	70 (77.8)	20 (22.2)		
**Number of presentations per individual**			0.403	0.864
1	448 (82.4)	96 (17.6)		
2	56 (77.8)	16 (22.2)		
3	17 (77.3)	5 (22.7)		
≥4	16 (94.1)	1 (5.9)		

Among the 655 unique speakers, 508 held confirmed Japanese medical licenses, of which, 425 were men (83.7%), and 83 were women (16.3%). We verified the licensure year for 500 speakers; however, eight remained unverified. The median licensure year was 1996 (IQR: 1987–2004) for men and 1997 (IQR: 1989–2005) for women, with no significant difference observed between both the genders (p =  0.301). Furthermore, gender distribution showed no significant association with the licensure year (Fisher’s exact test, p =  0.968; trend test, p =  0.463) (Table 3).

**Table 3:** Gender distribution of Japanese medical doctors at the JSA annual meetings, 2019–2024.

	Men no. (%)	Women no. (%)	p-value	
			Chi-square/Fisher’s exact test	Trend test
**Overall**	425 (83.7)	83 (16.3)		
**Year of medical licensure** ^a^			0.968	0.463
1965–1970	5 (100)	0 (0.0)		
1971–1980	23 (85.2)	4 (14.8)		
1981–1990	120 (85.1)	21 (14.9)		
1991–2000	125 (83.9)	24 (16.1)		
2001–2010	112 (81.8)	25 (18.2)		
2011–2020	35 (85.4)	6 (14.6)		

^a^The year of medical licensure was confirmed for 500, but not for eight individuals.

**Fig 3:**
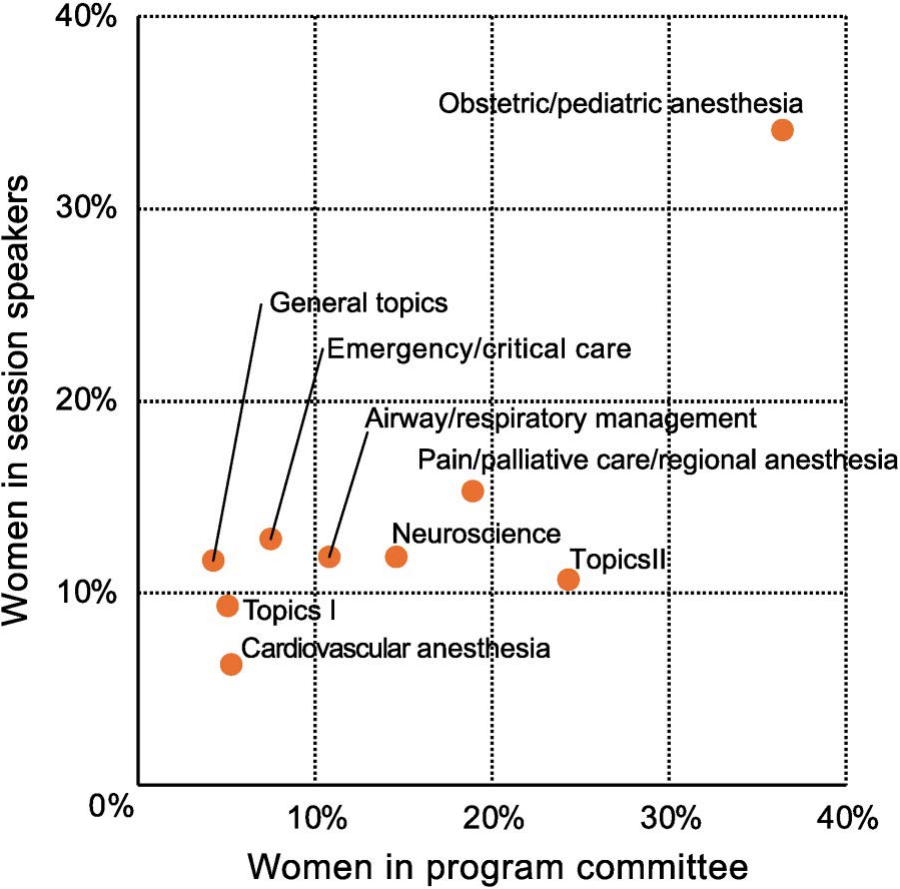
Relationship of women representation on program committees to session speakers.

Among the 437 program committee members at the JSA annual meetings during the six-year study period, 381 (87.2%) were men, and 56 (12.8%) were women. The proportion of women varied across committee positions (Table 4). Although the subgroups with a higher percentage of women on the program committee had more female speakers, this correlation was not statistically significant (correlation coefficient =  0.59, p =  0.092) (Fig 3).

**Table 4:** Gender distributions among program committee members.

	Men no. (%)	Women no. (%)
Chairperson	6 (100.0)	0 (0.0)
Vice-chairperson	7 (100.0)	0 (0.0)
Committee members	60 (92.3)	5 (7.7)
**Subspecialties**		
Obstetric/pediatric anesthesia	28 (63.6)	16 (36.4)
Anesthesia-related topics Ⅱ	28 (75.7)	9 (24.3)
Pain clinic, palliative care, regional anesthesia	30 (81.1)	7 (18.9)
Neuroscience and anesthesia	35 (85.4)	6 (14.6)
Airway and respiratory management	33 (89.2)	4 (10.8)
Emergency and critical. care medicine	37 (92.5)	3 (7.5)
Cardiovascular anesthesia	36 (94.7)	2 (5.3)
Anesthesia-related topics Ⅰ	37 (94.9)	2 (5.1)
General anesthesia topics	44 (95.7)	2 (4.3)

We analyzed 104 sessions from the JSA branch meetings between 2019 and 2023, excluding 2020 sessions due to meeting cancellations. These sessions included 176 speaker slots, of which 147 (83.5%) included men and 29 (16.5%) women. Gender distribution showed no significant differences between annual and branch meetings (p =  0.763) or between host regions (p =  0.984) (Table 5).

**Table 5:** Gender distribution of speaker slots at the JSA branch meetings, 2019–2023.

	Men no. (%)	Women no. (%)	p-value
**Overall**	147 (83.5)	29 (16.5)	
**Host region**			0.984
Hokkaido-Tohoku	24 (82.8)	5 (17.2)	
Kanto Koshinetsu-Tokyo	27 (79.4)	7 (20.6)	
Tokai-Hokuriku	20 (83.3)	4 (16.7)	
Kansai	38 (84.4)	7 (15.6)	
Chugoku-Shikoku	21 (87.5)	3 (12.5)	
Kyushu	17 (85.0)	3 (15.0)	

## Discussion

Our analysis of the gender distribution at JSA meetings revealed that women represented only 17.9% of speaker slots at annual and 16.5% at branch meetings. While direct comparisons are challenging owing to differing contexts, this representation appears lower than that at other anesthesia-related conferences: 25% at the ASA [5], 28.5% at the CAS [6], and 22–25% at the SCA [7]. The ASA and CAS data show that the speakers’ gender ratios align with their membership demographics [5,6]. A 2019 North American report indicated an increasing trend in women speakers at medical conferences, narrowing the gap in the proportion of practicing female physicians [14]. Considering that women comprised approximately 40% of JSA members during the study period, additional efforts are needed to enhance their representation at JSA meetings.

Gender disparity was especially pronounced in sessions with fewer speaker slots, suggesting that women had shorter speaking times than did men. This pattern has not been observed at meetings of the ASA or CAS [5,6]. Such imbalances can contribute to forming all-male panels—often criticized as “manels” [15–18]. At the JSA annual meetings, 44.3% of sessions were manels, an issue that needs to be addressed.

The pattern of gender disparity extended to subspecialties, with women well-represented in obstetric (36.8%) and pediatric anesthesia (31.8%), but significantly underrepresented in cardiovascular anesthesia (6.3%). Similar trends have been reported at annual CAS meetings [6]. These imbalances may reinforce gender stereotypes and perpetuate implicit biases, potentially misleading young physicians [19]. Future research should explore why women are more attracted to specific subspecialties and how to promote equal opportunities across all fields [6].

Our study uniquely investigated speakers’ medical licensure years; an approach uncommon in similar studies. Since the 2000s, the proportion of women in the JSA and among all Japanese physicians has increased [20]. We expected higher female representation among younger physicians but found no such generational differences. These results suggest that merely waiting for women to gain experience commensurate with speaking engagement is insufficient [21]; proactive interventions are necessary to enhance women’s representation.

Gender imbalance remains prevalent in the Japanese medical industry, as evidenced by women holding only 4.7% of medical school chief professorships in 2019 [22]. The Japan Inter-Society Liaison Association Committee for Equal Participation in Science and Engineering, comprising 111 academic societies (the JSA is not a member), regularly surveys women’s academic participation. Their 2023 survey revealed that while female speaker representation positively correlated with membership ratios across academic societies, the field of medicine lags behind other disciplines [23]. Thus, our findings mirror systemic gender inequalities in Japanese medicine rather than pertaining to only anesthesiology-specific specialty issues.

To enhance gender diversity, we must recognize the challenges women face [24]. Factors such as the “motherhood penalty,” stereotypical perceptions of male leadership, and impostor syndrome often discourage women from pursuing leadership roles [25–27]. Additionally, many women face the dual challenge of balancing their careers and family responsibilities [28]. Moreover, women are often undersponsored compared to men, making them less likely to be recommended as speakers by conference planners [29].

Organizational strategies are crucial beyond raising individual awareness. Academic societies should ensure that there is female representation on program committees, establish gender targets, and maintain data collection and reporting [2,8,30]. Such organizational approaches can help address the observed gender imbalance in speaker representation and program committee composition at JSA meetings. Women’s quotas aim to ensure equal opportunities for all qualified candidates, not to promote less-qualified women. Research on Swedish politics has demonstrated that such quotas enhance overall competence by reducing the proportion of mediocre male politicians [31].

This study had some limitations. First, gender identification was based on names or internet photos, which may not accurately reflect an individual’s gender identity and may not account for non-binary individuals. Second, we could not track those who received invitations to speak or declined to participate. Third, although we confirmed that the speakers were medical doctors, we could not verify their specialization in anesthesiology. Finally, we could not assess speakers’ academic or clinical accomplishments, which may have influenced their likelihood of receiving speaking invitations.

In conclusion, women accounted for only 17.9% of the speaker slots at the JSA annual meetings. Gender disparities were more pronounced in sessions with fewer speaker slots and specific subspecialties. Furthermore, despite the increasing proportion of women among JSA members, the representation of early-career female speakers has not improved. Gender imbalances in the composition of the program committee were also prominent. These findings underscore the necessity for proactive individual and organizational measures to promote gender diversity in Japanese anesthesiology.
